# Deficiency of RARα Suppresses Decidualization *via* Downregulating *CEBPB* Transcription in Women With Recurrent Implantation Failure

**DOI:** 10.3389/fendo.2022.753416

**Published:** 2022-05-19

**Authors:** Caiyi Huang, Qian Zhang, Tianxiang Ni, Tingting Zhou, Chunzi Lv, Yan Li, Junhao Yan, Zi-Jiang Chen

**Affiliations:** ^1^ Center for Reproductive Medicine, Ren Ji Hospital, School of Medicine, Shanghai Jiao Tong University, Shanghai, China; ^2^ Shanghai Key Laboratory for Assisted Reproduction and Reproductive Genetics, Shanghai Jiao Tong University, Shanghai, China; ^3^ Center for Reproductive Medicine, Cheeloo College of Medicine, Shandong University, Jinan, China; ^4^ National Research Center for Assisted Reproductive Technology and Reproductive Genetics, Shandong University, Jinan, China; ^5^ Key Laboratory of Reproductive Endocrinology of Ministry of Education, Shandong University, Jinan, China

**Keywords:** RARα, recurrent implantation failure (RIF), decidualization, *CEBPB*, endometria stromal cells

## Abstract

**Background:**

Recurrent implantation failure (RIF) is a disease associated with endometrial receptivity dysfunction. Retinoic acid receptor alpha (RARα) is an important protein in many biological processes, such as differentiation and development. However, the exact underlying mechanism whereby RARα affects RIF remains unknown. This study investigated RARα expression and its contribution in the mid-luteal phase endometria of patients with RIF.

**Methods:**

The expression levels of RARα and CCAAT/enhancer-binding protein (C/EBP) β in the endometria of the RIF and normal group were investigated using western blotting and immunohistochemistry. In *in vitro* experiments, immortal telomerase-transformed human endometrial stromal cells (T-HESCs) were incubated with medroxyprogesterone-17-acetate (MPA) and cyclic adenosine monophosphate (cAMP) for 4 days to induce decidualization. The expression levels of the decidualization markers prolactin (PRL) and insulin-like growth factor-binding protein-1 (IGFBP-1) were determined using quantitative polymerase chain reaction. RARα was knocked down using a small interfering RNA, and C/EBPβ was overexpressed from an adenoviral vector. The transcriptional regulation of *CEBPB* by RARα was determined by chromatin immunoprecipitation (ChIP) assay and luciferase assays.

**Results:**

We found that the expression levels of RARα decreased in the mid-luteal endometria of RIF patients. After 4 days of decidualization induction *in vitro*, RARα knockdown impaired the decidualization of T-HESCs and downregulated the expression of C/EBPβ. The restoration of C/EBPβ expression rescued the RARα knockdown-induced suppression of T-HESC decidualization. In ChIP analysis of lysates from decidualized T-HESCs, the *CEBPB* promoter region was enriched in chromatin fragments pulled down using an anti-RARα antibody. However, the relationship between *CEBPB* transcription and RARα expression levels was only observed when the decidualization of T-HESCs was induced by the addition of cAMP and MPA. To identify the binding site of RARα/retinoid X receptor α, we performed luciferase assays. Mutation of the predicted binding site in *CEBPB* (-2,009/-1,781) decreased the transcriptional activity of the reporter. To confirm this mechanism, the expression levels of C/EBPβ in the mid-luteal endometria of RIF patients were determined and found to decrease with decreased RARα expression levels.

**Conclusion:**

A deficiency of RARα expression in the mid-luteal endometrium inhibits decidualization due to the downregulation of *CEBPB* transcription. This is a potential mechanism contributing to RIF.

## Introduction

Embryo implantation is the first step of pregnancy. It is a complex but inefficient process. In the natural menstrual cycle, the chance of pregnancy is approximately 30% (1, 2). With advances in assisted reproductive techniques (ARTs), the implantation rate has reached approximately 70% per embryo transfer and cumulative implantation rate has reached 94.2%following 24-chromosome screening (3, 4). However, even with these improved techniques, certain diseases still prevent pregnancy at the first step. Recurrent implantation failure (RIF) is defined as failed implantation of more than three high-quality serially transferred embryos or more than 10 embryos in multiple transfers (5). Patients with RIF are infertile, even after continuous ART cycles.

The brief period during which embryo implantation is possible, also known as the window of implantation, coincides with the mid-luteal phase (6, 7). During this period, the endometrial environment is most suitable for embryo implantation. An embryo can only be successfully implanted during this state of endometrial receptivity (8). Many studies have shown that abnormal endometrial receptivity is associated with decidualization in response to hormone dysfunction (9, 10). Decidualization is a process unique to the endometrium, in which the appearance of the stromal cells changes in tandem with hormone fluctuations. Stromal cells initially have a fibroblast-like appearance in the proliferative phase. Subsequently, in the secretory phrase, the nuclei become rounded, with increased numbers of nucleoli, and the cytoplasm expands with the accumulation of glycogen and lipid droplets (11). These changes are related to the orchestration of endometrial receptivity, embryo selection, and embryo-maternal crosstalk during implantation (12).

Retinoic acid receptor alpha (RARα) participates in many biological processes, such as differentiation and development (13). RARα can bind to retinoid X receptors (RXRs) to form an RXR/RAR heterodimer, which then binds to DNA, thereby regulating downstream gene expression (14). RAR expression levels change throughout the menstrual period. Previous studies have found that the protein levels of RARs in the nuclei of stromal cells increase in the proliferation phase and then decrease in the secretory phrase (15–17). These results suggest that RARs may predominantly function in the proliferative phase. Subsequent studies have mainly focused on the relationship between RARs and estrogen or have aimed to determine the mechanism whereby RARs affect endometrial proliferative disorders, such as endometriosis, endometrial hyperplasia, or endometrial carcinoma (18, 19). However, the function of RARα in the endometrial secretory phase remains unclear. The role of RARα in secretory phase disorders, such as implantation failure and pregnancy loss, has not yet been investigated. Therefore, in this study, we determined the expression level of RARα in endometrial tissues of RIF patients and investigated the effect of RARα on decidualization.

## Materials and Methods

### Ethics Statement

This study was approved by the Institutional Review Board of the Center for Reproductive Medicine, Shandong University, China. Written consent was obtained from all participants.

### Patients and Endometrial Samples

All participants were recruited from Shandong University Affiliated Hospital for Reproductive Medicine. The criteria for enrolment in the RIF group were: 1) implantation failure of more than three serially transferred high-quality embryos or more than 10 embryos from multiple transfers; 2) a normal karyotype for both individuals in the couple; 3) a maternal age < 40 years; 4) no uterine abnormalities, spontaneous abortions, or autoimmune diseases; and 5) a regular menstrual cycle (21–35 days) with no steroid hormone use for more than 1 month. Participants were included in the control group if they had conceived within the first three high-quality embryo transfers or had a history of successful pregnancy before *in vitro* fertilization (IVF) or intracytoplasmic sperm injection and met inclusion criteria 2–5 for the RIF group. Nineteen RIF patients and 13 control IVF patients were recruited. The characteristics of the control group and the RIF patients are presented in **Table 1**.

**Table 1:** The basal characteristic of control group and RIF patients.

	Control (n=13)	RIF (n=19)	p-value
Age (year)	31.38 ± 5.19	34.21 ± 3.89	0.088
BMI (cm/kg^2^)	24.16 ± 4.14	22.47 ± 2.99	0.104
Basic FSH(IU/L)	6.68 ± 1.06	6.88 ± 1.14	0.623
Basic LH (IU/L)	5.34 ± 2.11	5.89 ± 3.89	0.642
Right AFC(n)	7.31 ± 3.04	10.00 ± 6.79	0.192
Left AFC(n)	8.00 ± 3.24	8.58 ± 4.87	0.710

All data are showed as mean ± SD; the comparation of two group (Control/RIF) were used Student’s t-test.

Endometrial biopsies were performed 5–7 days after ovulation during a natural menstrual cycle for patients and control subjects. Samples were immediately snap-frozen in liquid nitrogen and stored at -80°C for subsequent processing (RNA isolation and western blotting). Samples for immunohistochemistry (IHC) were fixed in 4% paraformaldehyde for 24 hours.

### RNA Isolation and Quantitative Polymerase Chain Reaction

Total RNA was extracted from endometrial samples or telomerase-transformed human endometrial stromal cells (T-HESCs) using TRIzol reagent (TaKaRa, Dalian, China) following the manufacturer’s protocol. RNA was reverse transcribed into cDNA using a reverse transcription kit (TaKaRa). Gene expression levels were determined by quantitative (q) polymerase chain reaction (PCR) using SYBR Green chemistry (TaKaRa) and a LightCycler^®^ 480 instrument (Roche, Basel, Switzerland). Oligonucleotide-specific primer sequences were designed using the National Center for Biotechnology Information primer design tool (https://www.ncbi.nlm.nih.gov/tools/primer-blast/). The PCR primers were as follows: *RARA* forward, 5′-GGGCAAATACACTACGAACAACA-3′ and reverse, 5′-CTCCACAGTCTTAATGATGCACT-3′; *CEBPB* forward, 5′-CGACGAGTACAAGATCCGGC-3′ and reverse, 5′-TGCTTGAACAAGTTCCGCAG-3′; prolactin (*PRL*) forward, 5′-CATATTGCGATCCTGGAATGAG-3′ and reverse, 5′-GATGAACCTGGCTGACTATCA-3′; insulin-like growth factor-binding protein 1 (*IGFBP1*) forward, 5′-GGCACAGGAGACATCAGGAGAA-3′ and reverse, 5′-GATGAACCTGGCTGACTATCA-3′ and glyceraldehyde 3-phosphate dehydrogenase (*GAPDH*) forward, 5′-GGAGCGAGATCCCTCCAAAAT-3′ and reverse, 5′- GGCTGTTGTCATACTTCTCATGG-3′. mRNA expression levels were normalized to *GAPDH* expression levels.

### Western Blotting Analysis of Protein Expression

Total proteins were extracted from T-HESCs using a lysis buffer (Beyotime, Shanghai, China) supplemented with a protease inhibitor cocktail (1:100 dilution; CWBio, Beijing, China). All extracted proteins were heated at 100°C for 10 min and then stored at -80°C. The proteins were separated on a 10% sodium dodecyl sulfate-polyacrylamide gel electrophoresis system and transferred to polyvinylidene difluoride membranes (Millipore, Burlington, MA, USA). The membranes were blocked with 5% nonfat milk in Tris-buffered saline with Tween-20 and incubated with the following primary antibodies: anti-RARα (1:1,000 dilution; 62294, lot #1, Cell Signaling Technology, Danvers, MA, USA), anti-C/EBPβ (1:150 dilution; SC-7962, lot#12017, Santa Cruz Biotechnology, Dallas, TX, USA) and anti-GAPDH (1:5,000 dilution; SA00001-1/SA00001-2, lot#20000275/20000311, Proteintech, Wuhan, China). The membranes were then incubated with horseradish peroxidase-conjugated secondary antibodies (1:5,000 dilution, Proteintech). Western blotting was then performed, and the labeled protein bands were developed using HRP (Millipore). The intensity of the bands was determined using Image Lab software (Bio-Rad, Hercules, CA, USA).

### Immunohistochemistry

Endometrial tissues were obtained from control IVF patients and RIF patients. Tissue samples were fixed in 4% paraformaldehyde for 24 hours, dehydrated, embedded in paraffin, and stored at -20°C. Tissue sections were heated at 37°C overnight and then de-waxed, hydrated through a graded alcohol series (100%, 95%, 75%), and washed with distilled water. After immersing the sections in an ethylenediaminetetraacetic acid solution and boiling for 15 min for antigen retrieval, they were incubated in 3% H_2_O_2_ for 15 min to block endogenous peroxidase activity and then blocked with bovine serum albumin for 1 hour. Tissue sections were incubated in a moist chamber overnight at 4°C with an anti-RARα primary antibody (1:50 dilution, 62294, lot #1, Cell Signaling Technology) or an anti-C/EBPβ primary antibody (1:50 dilution; SC-7962, lot#12017, Santa Cruz Biotechnology, Dallas, TX, USA). After incubation at room temperature for 20 min with a secondary antibody (ZSGB-bio, Beijing, China), the signal was developed using a diaminobenzidine kit (ZSGB-bio). Negative control is incubated with antibody diluent, without the primary antibody included, and then followed the same procedure above. Images were captured at the same magnification and under the same exposure conditions for each sample. Representative images of all staining intensities were captured at random. IHC results were quantified using the H-score method, in which the percentage of positive cells (0–100%) and staining intensity (0–3+) were considered. A final score was calculated on a continuous scale from 0 to 300 using the following formula: H-sore =1 × (% of cells 1+) + 2 × (% of cells 2+) + 3 × (% of cells 3+).

### Cell Culture and *In Vitro* Decidualization

Immortal T-HESCs were cultured in phenol red-free Dulbecco’s modified Eagle’s medium (DMEM)/F12 containing glutamine (Gibco, Grand Island, NY, USA), 1% penicillin-streptomycin (HyClone, Logan, UT, USA), 1% NaHCO_3_ and 10% dextran-coated charcoal-stripped fetal bovine serum (C-FBS; Biological Industries, Beit HaEmek, Israel) at 37°C and 5% CO_2_. To induce *in vitro* decidualization, the cells were incubated for 4 days with differential medium consisting of phenol red-free DMEM/F12 with 2% C-FBS, 0.5 mM 8-bromo-adenosine-3′,5′-cyclic monophosphate (cAMP; Sigma-Aldrich, St. Louis, MO, USA) and 10^-6^ M medroxyprogesterone-17-acetate (MPA, Sigma-Aldrich). The differential medium was changed every 48 hours. The PRL and IGFBP-1, which were classic decidual markers, were used to assess the differentiation status of HESCs in culture.

### siRNA Transfection and Adenovirus Transduction

T-HESCs were seeded in antibiotic-free medium in six-well plates; after reaching 60% confluence, they were transfected with a small interfering RNA (siRNA) specific for *RARA* (si-RNA *RARA*, GenePharma, Shanghai, China) or with a negative control (NC) siRNA (GenePharma) for 24 hours using Lipofectamine 3000 reagent (Invitrogen, Carlsbad, CA, USA). All procedures were performed according to the manufacturer’s instructions. For the transfection of siRNA *RARA*, 40 pmol of siRNA and 3.5 µL of Lipofectamine 3000 were diluted with 100 µL of reduced-serum medium (Opti-MEM, Gibco) per mL. Both transfection mixtures were incubated for 15 min at room temperature and added dropwise to each well. After 6 hours, the transfected cells were washed with phosphate-buffered saline (PBS), and *in vitro* decidualization was induced as described above for 4 days. T-HESCs in the undifferentiated group were incubated with non-selective medium for 4 days. In a rescue experiment, an adenoviral vector engineered to overexpress *CEBPB* (Vigene, Jinan, China) was transduced into T-HESCs (volume ratio: 1:5,000) 24 hours after transfection with siRNA *RARA* to restore *CEBPB* expression. The cells were then incubated in differential or non-selective medium. Twenty-four hours after adenovirus transduction, the differential or non-selective medium was removed, and the cells were washed with PBS and incubated in differential medium for another 48 hours. The siRNA sequences were as follows: *RARA*, forward 5′-GGGUGAUCACGCUGAAGAUTT-3′ and reverse, 5′-AUCUUCAGCGUGAUCACCCTT-3′.

### Plasmid Transfection

RARα expression plasmid (pET-*RARA*, Vigene, Jinan, China) were used for overexpression. For the transfection of pET-*RARA*, 2.5ug plasmid and 5 µL of Lipofectamine 3000 (Invitrogen, Carlsbad, CA, USA) and 5ul P3000 were diluted with 100 µL of reduced-serum medium (Opti-MEM, Gibco) per ml. All procedures were performed according to the manufacturer’s instructions. The other steps of experiment were following the “siRNA transfection”.

### Chromatin Immunoprecipitation

The RARα binding site was predicted using the transcription factor binding profile database JASPAR (http://jaspar.genereg.net/). The 2200 nucleotides upstream of the coding sequence were selected as the *CEBPB* promoter region for input into JASPAR. The primers used for chromatin immunoprecipitation (ChIP)-PCR were designed using the tool at http://pcrsuite.cse.ucsc.edu/ (20). The sequences of the *CEBPB* (-2,009/-1781) primers were as follows: forward, 5′-AAGGTCAGGGAGGGTTTCC-3′ and reverse, 5′-CCAGCCACCATTATCCTAGC-3′. T-HESCs were exposed to differential medium when cell confluence reached 80%. After 4 days of incubation, ChIP was performed using an EZ-Magna ChIP™ A/G Chromatin Immunoprecipitation Kit (17-10086, Millipore) according to the manufacturer’s instructions. Undifferentiated T-HESCs were incubated in standard non-selective medium for 4 days; when they reached 80% confluence, they were used in ChIP experiments. In brief, T-HESCs were fixed with 1% formaldehyde at room temperature for 10 min. The cells were lysed with cell and nuclear lysis buffer, after which sonication was performed to shear the chromatin into 300–1,000 bp fragments. The chromatin fragments were immunoprecipitated with an anti-RARα antibody (Cell Signaling Technology) and IgG (Abcam, Cambridge, UK). Finally, the precipitates were collected and analyzed by ChIP-PCR and ChIP-qPCR. After 32 cycles of PCR, the amplification products were analyzed by 1.5% agarose gel electrophoresis, and the level of chromatin enrichment was quantified.

### Luciferase Reporter Assay

Human embryonic kidney (HEK293) cells were cultured in DMEM (Gibco) with 10% fetal bovine serum (Biological Industries) and 1% penicillin-streptomycin (HyClone) in a 37°C incubator with 5% CO_2_.

Firefly luciferase reporter constructs were designed with the upstream -2,200 *CEBPB* promoter region (pGL3-*CEBPB*) and with a mutated predicted binding site (-2,009/-1,781) in the *CEBPB* promoter (pGL3-*CEBPB*-mutant). An RARα expression plasmid (pET-*RARA*, Vigene, Jinan, China) and the reporter plasmids (pGL3-*CEBPB* or pGL3-*CEBPB*-mutant) were used to transfect HEK293 cells at a 1:3 ratio with Lipofectamine 3000 (Invitrogen). All procedures were performed according to the manufacturer’s instructions. Forty-eight hours after transfection, luciferase activities were measured using a dual-luciferase assay (Promega). Firefly/*Renilla* fluorescence ratios were calculated to determine the role of RARα in the regulation of *CEBPB* promoter activity.

### Data Analysis

The data presented here are representative of three or more biological replicates. The data are presented as the mean ± standard error of the mean. Significant differences between two groups were analyzed by Student’s t-test, using Prism Version 7 (GraphPad, San Diego, CA, USA). *P*-values < 0.05 were considered to be statistically significant.

## Results

### Expression Levels of RARα Decreased in Mid-Luteal Phase Endometria of RIF Patients

**Figure 1:**
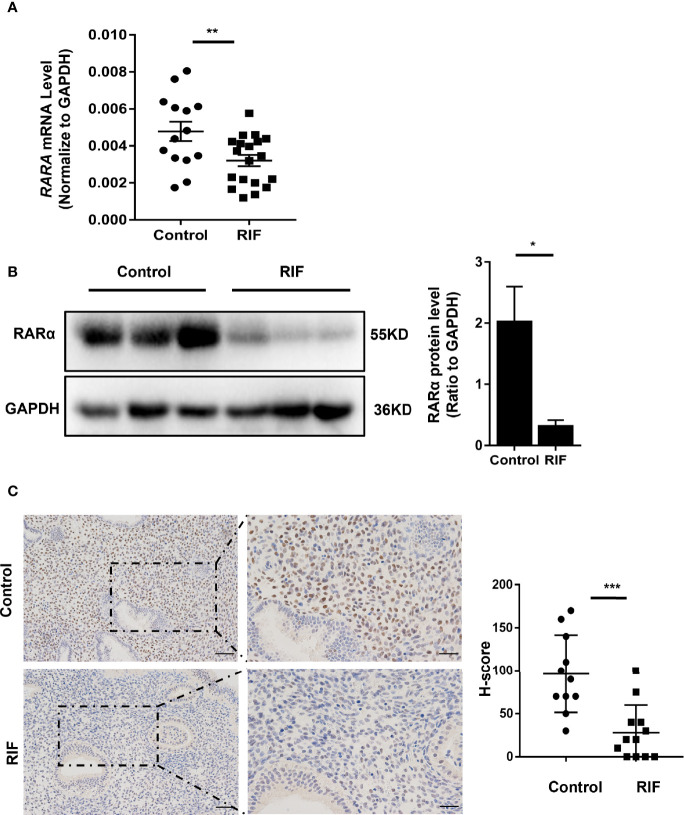
Expression of RARα in endometrium of patients with RIF. **(A)** RARα mRNA expression in RIF (n=19) and normal women (n=13). **(B)** Representative Western blot and quantification of RARα levels in RIF (n=3) and control patients (n=3). **(C)** Localization of RARα in endometrium of patients with RIF (n=12) and normal women (n=11) (Scale bar, left: 50μm, right: 25μm). Protein and mRNA expression levels are normalized to GAPDH expression. The comparison of RIF and normal group in IHC were used H-score. All data are showed as mean ± SEM. The statistic difference between two groups was determined by Student’s t-test, *P < 0.05, **P < 0.01, ***P < 0.001.

The basal characteristics of the control and RIF groups are listed in **Table 1**. There were no significant differences in maternal age, body mass index, basal antral follicle count, basal follicle-stimulating hormone concentration, or luteinizing hormone concentration between the RIF and control groups. As shown in **Figures 1A, B**, *RARA* mRNA and RARα protein expression levels were significantly lower in the mid-luteal phase endometria of RIF patients than control participants (*P* = 0.010 and *P* =0.04). IHC analysis (**Figure 1C**) showed high levels of RARα protein localized in the nuclei of endometrial stromal cells. These results implied that RARα mainly plays its role in the nuclei of stromal cells in the mid-luteal phase. IHC analysis also showed that RARα protein levels were clearly decreased in the nuclei of stromal cells in the endometria of RIF patients compared with the endometria of control participants. The H-score of RARα in the nuclei of endometrial stromal cells was lower in the RIF group than the control group (*P* < 0.001).

### RARα Knockdown Impaired Decidualization and RARα Overexpression Enhanced Decidualization in Decidualized T-HESCs

**Figure 2:**
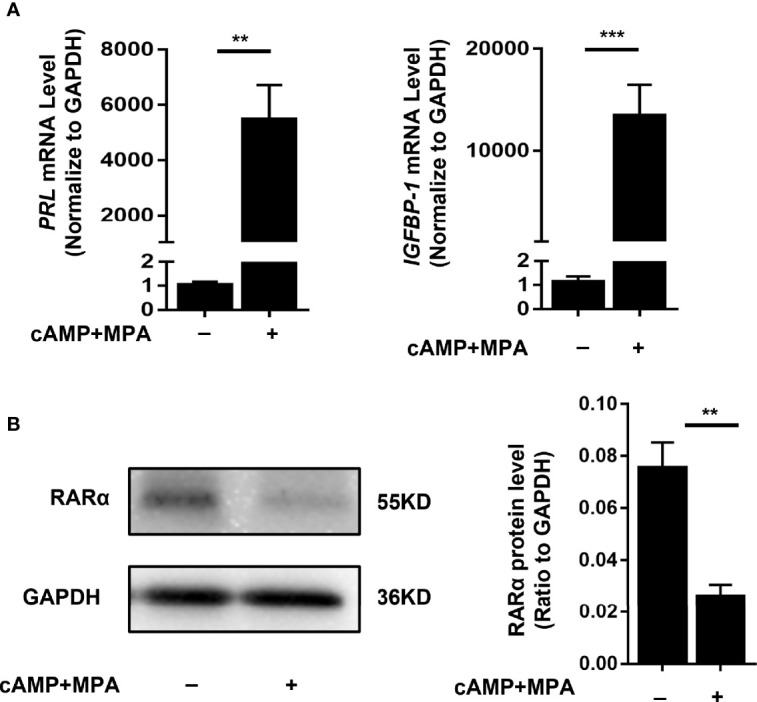
Stimulation of decidualization in T-HESCs. **(A)** PRL and IGFBP-1 mRNA expression of T-HESCs after 4-days induction of decidualization with cAMP and MPA. **(B)** The RARα expression after 4-days induction of decidualization with cAMP and MPA. Expression of mRNA and protein levels are normalized to GAPDH expression. All data are showed as mean ± SEM. The statistic difference between two groups was determined by Student’s t-test, **P < 0.01, ***P < 0.001.

**Figure 3:**
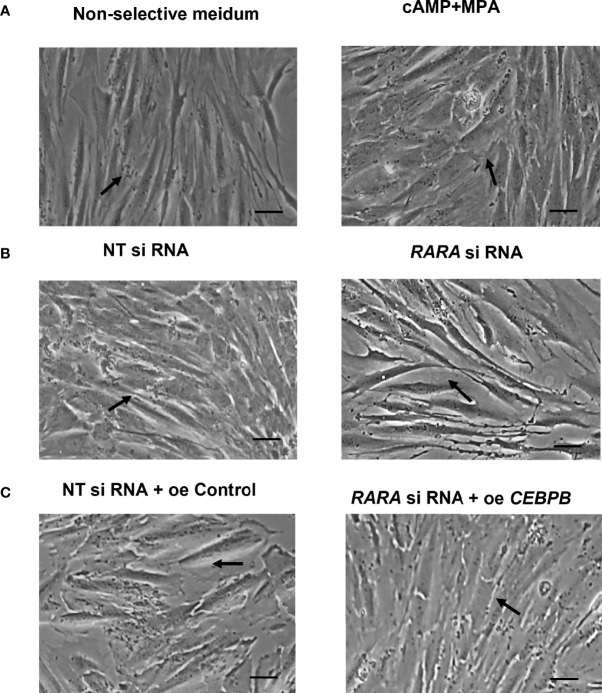
**(A)** Morphology of T-ESCs after 4-days stimulation of decidualization with cAMP and MPA (Scale bar, left: 100μm). **(B)** Cellular morphology of decidualized T-HESCs with RARα knockdown (Scale bar, left: 100μm). **(C)** Cellular morphology of decidualized T-HESCs with RARα knockdown and C/EBPβ over-expression (Scale bar, left: 100μm).

To determine whether decreased RARα expression levels affected the decidualization process, we transfected decidualized T-HESCs with siRNA *RARA* or an NC siRNA. T-HESCs were treated with cAMP and MPA for 4 days to induce decidualization. The induction of decidualization for 4 days was successful. The PRL and IGFBP-1, two classic decidual marker, were detected, and the morphology change of T-HESCs were recorded (**Figures 2A**, **3A**). The *PRL* and *IGFBP-1*mRNA level were significantly increased (*P*<0.01 and *P*<0.001). After 4-days induction, the expression of RARα were significantly decreased (**Figure 2B**, *P*=0.009).

**Figure 4:**
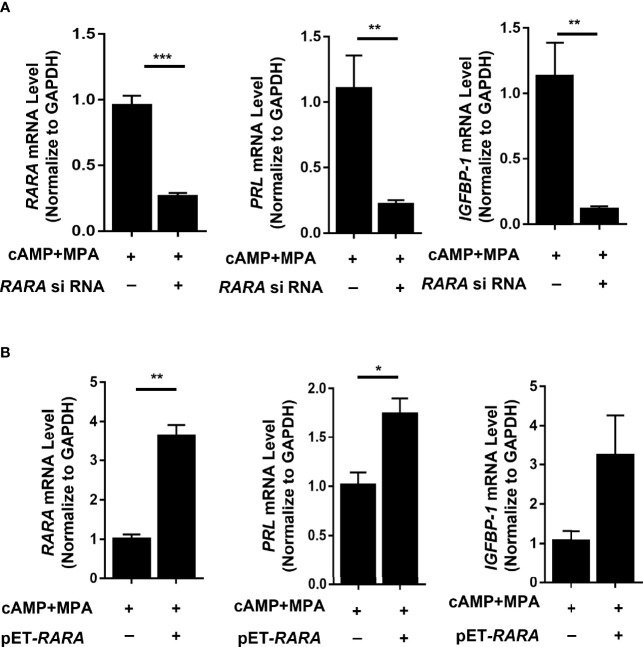
RARα knockdown and RARα overexpression in decidualized T-HESCs. **(A)** PRL, IGFBP1 mRNA expression level in T-HESCs with RARα knockdown. **(B)** PRL, IGFBP1 mRNA expression level in T-HESCs with RARα overexpression. Expression levels of mRNA are normalized to GAPDH expression. All data are shown as means ± SEMs. Statistically significant differences between two groups were determined by Student’s t-test, *P < 0.05, **P < 0.01, ***P < 0.001.

After induction, *PRL* and *IGFBP1* mRNA levels were significantly decreased (*P* = 0.008 and *P* = 0.004, respectively) in the decidualized T-HESCs with RARα knocked down (**Figure 4A**). The consequent cellular morphology is shown in **Figure 3B**. Without RARα knockdown, the T-HESCs transformed into large, round decidual cells after stimulation with decidualization-inducing conditions; however, when RARα was knocked down, decidualization was inhibited and the T-HESCs remained fibroblast-like.

The RARα expression decreased after 4-days induction, however, RARα knockdown impaired decidualization. To further explore the role of RARα in decidualization, plasmid of *RARA* was used to overexpressing RARα. After induction, PRL mRNA levels were significantly increased (*P*=0.022) and IGFBP-1 mRNA levels were clearly higher in the decidualized T-HESCs with RARα overexpression (**Figure 4B**). Results of RARα knockdown and overexpression demonstrated that, although its expression decreased compared with the proliferative status, certain amount of RARα expression is crucial in decidualization.

### RARα Knockdown Downregulated *CEBPB* Levels in Decidualized T-HESCs

**Figure 5:**
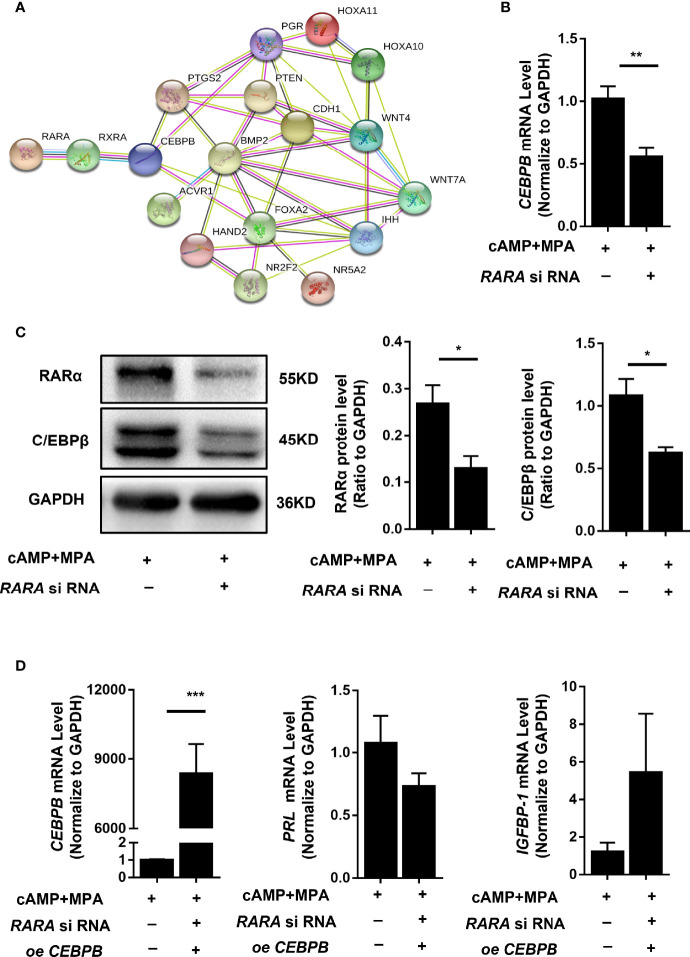
RARα knockdown downregulated C/EBPβ expression and restoration of C/EBPβ expression rescued the suppressed decidualization of T-HESCs with RARα knockdown.in decidualized T-HESCs. **(A)** Results of predicted protein–protein interactions between critical proteins during decidualization. **(B)** C/EBPβ mRNA expression levels in RARα-knockdown decidualized T-HESCs. **(C)** C/EBPβ protein expression levels in RARα-knockdown decidualized T-HESCs. **(D)** PRL and IGFBP-1 mRNA expression after overexpressing C/EBPβ in decidualization of T-HESCs with RARα knockdown. Protein and mRNA expression levels are normalized to GAPDH expression. All data are shown as means ± SEMs. Statistically significant differences between two groups were determined by Student’s t-test, *P < 0.05, **P < 0.01, ***P < 0.001.

RARα participates in numerous physiological processes by forming heterodimers with RXR and regulating the expression of a series of genes. RARα may therefore affect decidualization by regulating the expression of a determinant gene. We used the protein-protein network database STRING (https://string-db.org/) to predict protein interactions, using crucial genes for decidualization as the STRING inputs (12). As shown in **Figure 5A**, a potential interaction was found between RAR/RXR and *CEBPB*.

*CEBPB* is a crucial gene for embryo implantation and decidualization (21). *CEBPB* mRNA (*P* = 0.006) and C/EBPβ protein expression levels were significantly decreased in T-HESCs subjected to RARα knockdown and 4 days of *in vitro* decidualization (**Figures 5B, C**). This result implied that RARα might influenced decidualization *via* regulating C/EBPβ transcription.

### Restoration of C/EBPβ Reversed the Suppressed Decidualization in T-HESCs With RARα Knocked Down

To further determine the relationship between C/EBPβ and RARα during decidualization, we restored C/EBPβ expression in T-HESCs with RARα knocked down. C/EBPβ overexpression rescued the decidualization of these T-HESCs (**Figure 5D**). After C/EBPβ overexpression, the transcription levels of *PRL* and *IGFBP-1* were increased in T-HESCs with RARα knocked down compared with control cells. The consequent morphology of C/EBPβ-overexpressing T-HESCs with RARα knocked down is shown in **Figure 3C**. Without C/EBPβ overexpression, the T-HESCs with RARα knocked down transformed into larger, rounder cells compared with those shown in **Figure 3B**, which were more decidual-like after stimulation with decidualization-inducing conditions.

### RARα Directly Regulated *CEBPB* Transcription in Decidualized T-HESCs

**Figure 6:**
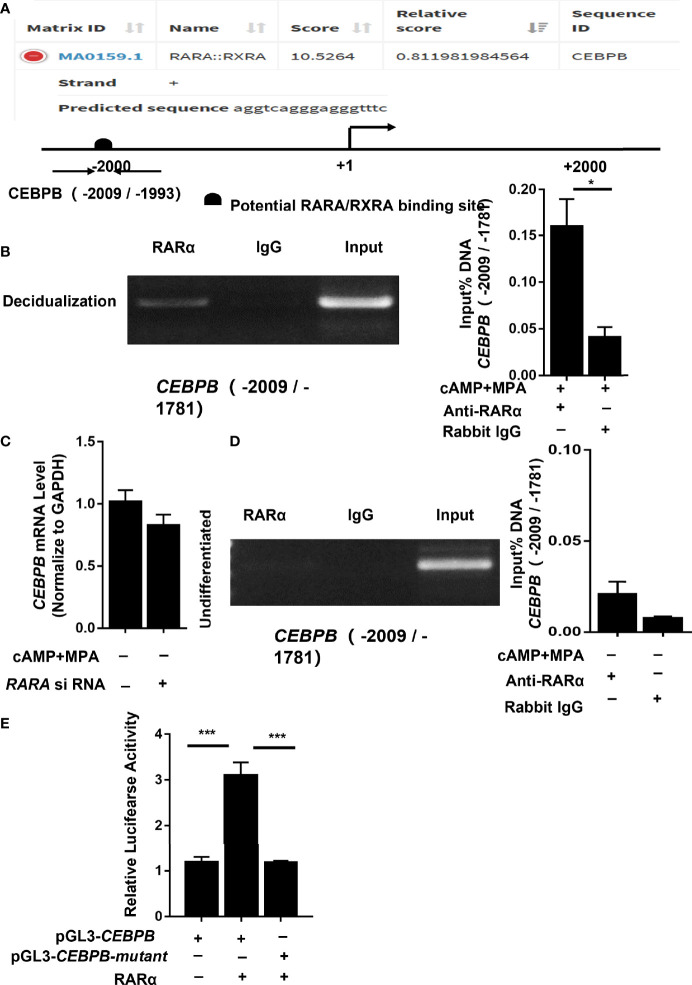
RARα directly regulated *CEBPB* transcription in decidualized T-HESCs. **(A)** *In silico* (JASPAR)-predicted binding site between RARα and the CEBPB upstream -2200 promoter region. **(B)** Results of ChIP experiment performed in decidualized T-HESCs. **(C)** C/EBPβ mRNA expression levels in RARα-knockdown T-HESCs absent MPA and cAMP. **(D)** Results of ChIP experiment performed in T-HESCs absent MPA and cAMP. **(E)** Result of luciferase reported assay for mutation of predicted binding site (-2,009/-1,781) in the CEBPB promoter. Chromatin was immunoprecipitated with anti- RARα. ChIP-qPCR results are normalized to the input DNA. Expression of mRNA levels are normalized to GAPDH expression. Firefly/Renilla fluorescence ratios were calculated to determine the promoter activity. All data are shown as means ± SEMs. Statistically significant differences between two groups were determined by Student’s t-test. *P < 0.05, ***P < 0.001.

To determine whether RARα regulates *CEBPB* directly, the *CEBPB* -2,200 nucleotide region was input into JASPAR for putative binding site prediction (**Figure 6A**). The results indicated a potential RARα/RXRα binding site in the region of *CEBPB* (-2,009/-1,993). To confirm whether RARα was capable of binding to this region, we performed ChIP assays on T-HESCs that had been cultured with or without cAMP and MPA for 4 days, using primers specific for *CEBPB* (-2,009/-1,781). PCR analysis of the ChIP precipitates showed that RARα bound to and enriched the predicted region of *CEBPB* from a lysate of decidualized T-HESCs (**Figure 6B**). To further calculate the fold enrichment of the *CEBPB* promoter region bound to the pulled-down chromatin, ChIP-qPCR was performed, and the results were normalized to the amount of input DNA. As **Figure 6B** shows, compared with IgG alone, an anti-RARα antibody significantly enriched the *CEBPB* promoter region (*P* = 0.01) pulled down from the lysate of decidualized T-HESCs.

### *RARA* Knockdown Did Not Decrease *CEBPB* Transcription in Undifferentiated T-HESCs

To further explore the relationship between RARα and *CEBPB* and to identify the stimulatory agent that influences *CEBPB* transcription, we detected changes in *CEBPB* mRNA levels in non-selective medium. In the absence of culture medium containing cAMP and MPA, the *CEBPB* mRNA expression levels did not change significantly (*P* = 0.171) after RARα knockdown (**Figure 6C**). In ChIP experiments (**Figure 6D**) without cAMP and MPA stimulation, the promoter region of *CEBPB* (-2,009/-1,781) was not pulled down by an anti-RARα antibody in undifferentiated T-HESCs (*P* = 0.109).

### Mutation of the Predicted Binding Site, *CEBPB* (-2,009/-1,781), Decreased Transcriptional Activity

To further determine the binding site of RARα/RXRα, we used a plasmid directing the expression of RARα and the firefly luciferase reporter constructs pGL3-CEBPB and pGL3-CEBPB-mutant. A luciferase reporter assay was used to monitor the transcriptional activity of the 2,200 upstream nucleotides of the *CEBPB* promoter region and the *CEBPB* (-2,009/-1,781) mutant in HEK 293 cells overexpressing RARα protein. RARα protein was expressed in transfected cells, and transcriptional activity was then measured from a responsive reporter. As shown in **Figure 6E**, cells transfected with pGL3-CEBPB showed increased transcription of the luciferase gene compared with those transfected with pGL3-CEBPB-mutant(*P*<0.001).

### Expression Levels of C/EBPβ Decreased in the Mid-Luteal Phase Endometria of Patients With RIF

**Figure 7:**
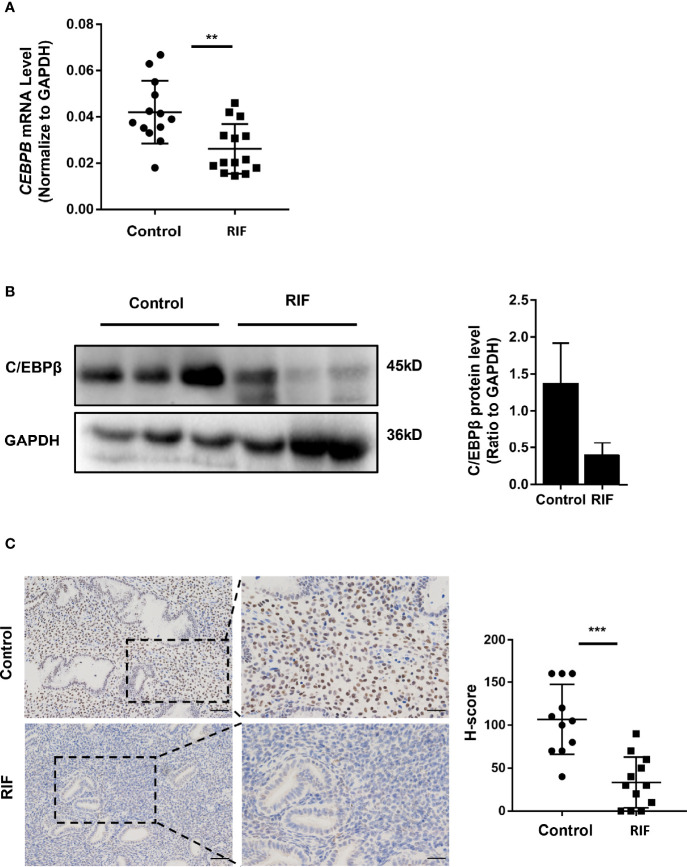
Expression of C/EBPβ in endometrium of patients with RIF. **(A)** C/EBPβ mRNA expression in RIF (n=14) and normal women (n=13). **(B)** Representative Western blot and quantification of C/EBPβ in RIF (n=3) and normal women (n=3). **(C)** Localization of C/EBPβ in endometrium of RIF patients(n=12) and normal women (n=11) (Scale bar, left: 50μm, right: 25μm). The comparison of RIF and normal group in IHC were used H-score. All data are showed as mean ± SEM. The statistic difference between two groups was determined by Student’s t-test, **P < 0.01, ***P < 0.001.

To determine whether the expression of C/EBPβ was affected by decreased RARα levels in the mid-luteal phase endometria of patients with RIF, we measured C/EBPβ protein levels in endometrial samples of patients with RIF (the same samples used for RARα measurement). As presented in **Figures 7A, B**, C/EBPβ mRNA were significantly decreased (*P*=0.0024) and protein levels were clearly lower in patients with RIF than in control IVF patients. IHC analysis (**Figure 7C**) showed that C/EBPβ was mainly localized in the nuclei of endometrial stromal cells. C/EBPβ levels in stromal cell nuclei clearly decreased in the mid-luteal phase endometria of patients with RIF. The H-score of C/EBPβ in the nuclei of endometrial stromal cells was lower in the RIF group than the control group (*P* < 0.001).

## Discussion

We found that RARα expression levels decreased in the mid-luteal phase endometria of patients with a history of RIF. This result was the opposite of what we had speculated. Previous findings have shown that during the menstrual cycle, RARα expression levels increase during the proliferative phase, then decrease in the secretory phase (15, 16). Given these findings, we initially hypothesized that RARα may be expressed at higher levels in the secretory phase endometria of RIF patients than normal control subjects. However, expression levels of RARα decreased in mid-luteal phase endometria of RIF patients.

To explore the function of RARα, we transfected siRNA specific for *RARA* into T-HESCs and then induced decidualization. After RARα knockdown, we found that the decidualization of T-HESCs was compromised. This result was consistent with the result of a recent study, which found that suppressing *RARA* expression levels in primary T-HESCs inhibits their decidualization (22). Ochiai et al. also demonstrated that resveratrol interferes with the decidualization of T-HESCs by downregulating retinoic acid-binding protein 2 and RAR expression levels (22). Previous studies have revealed the crucial roles of retinoid and retinoic acid (RA) signaling pathways in decidualization and investigated the associated mechanisms (23, 24). RARα, in particular, has been studied as part of the RA signaling pathway. Decidualization silences RA signaling by downregulating the expression of key cytoplasmic binding proteins and upregulating the expression of retinoid metabolism-related enzymes (23). Although RARα expression decreases during decidualization, this decrease has also been shown to suppress decidualization (22). A new study utilizes mice expressing dominant-negative form of RARα have confirmed that RA signaling is fundamental to decidualization., and deficiency of RAR-signaling leads to reduced follistatin and aberrant activin signaling (25). While previous studies have provided insights into how RA and the decidualization process suppress RA signaling (22–24), there are few studies investigating how decreased RARα expression levels impair decidualization. Considering that the characteristic function of the RAR/RXR heterodimer is the regulation of downstream gene expression, we conjectured that RARα influenced the decidualization process in fertility disorders by acting as a transcription factor.

After a series of protein interaction predictions (**Figure 5A**), we identified *CEBPB* as a candidate downstream target gene of RARα. C/EBPβ is crucial during implantation and decidualization. During decidualization in mice, C/EBPβ is rapidly induced and highly concentrated in stromal cells at blastocyst attachment sites. Knocking out *Cebpb* in female mice results in infertility, with a complete lack of decidual formation (21). The loss of C/EBPβ expression has been shown to impair the differentiation of primary T-HESCs in response to progesterone and cAMP (26).We examined *CEBPB* expression in decidualized T-HESCs with RARα knocked down. *CEBPB* mRNA and C/EBPβ protein levels were significantly decreased after knocking down RARα expression in decidualized T-HESCs. Previous studies have reported that *CEBPB* is the major RARα-responsive gene in the CEBP family and is necessary for the expression of genes involved in the functions of myeloid and mouse embryonic fibroblasts (27, 28). Our results demonstrated that *CEBPB* also responds to RARα in decidualized endometrial stromal cells. RARα knockdown in decidualized T-HESCs led to the downregulation of *CEBPB* expression. However, the restoration of C/EBPβ expression also restored the decidualization of T-HESCs with RARα knocked down. In the process of decidualization, the relationship between RARα and *CEBPB* remains unclear. RARα often forms heterodimers with RXRs, and these heterodimers participate in gene regulation. Predictions derived using JASPAR also suggested a potential relationship between RARα and *CEBPB*, wherein RARα forms a heterodimer with RXRα to regulate *CEBPB* expression. ChIP assays revealed that RARα was capable of binding to the *CEBPB* promoter region (-2,009/-1,781) in decidualized T-HESCs.

In cyclic menstruation, C/EBPβ levels markedly increase in stromal cell nuclei beginning at approximately cycle day 20, which coincides with the start of the mid-luteal phase (29). However, the levels of RARα in the nuclei of stromal cells increase in the proliferative phase and decrease in the secretory phase. In *in vitro* studies, RARα expression levels have been shown to decrease (30), which was consistent with our result (**Figure 2B**), while C/EBPβ expression levels have been shown to increase during *in vitro* decidualization (21). Changes in RARα and C/EBPβ expression levels appear to be discordant between *in vivo* and *in vitro* studies. To investigate this discordance, we determined *CEBPB* expression levels in T-HESCs cultured in non-selective medium and with RARα knocked down. In the absence of cAMP and MPA stimulation, *CEBPB* expression levels remained stable in the RARα-knockdown group compared with the NC group. In ChIP experiments, RARα did not bind to the *CEBPB* promoter region (-2,009/-1,781) in undifferentiated T-HESCs. These results partly explain why the expression patterns of RARα and C/EBPβ show contrasting trends in the mid-luteal phase and throughout decidualization *in vitro*. Based on these findings, we propose that RARα participates in the transcriptional regulation of *CEBPB* in the mid-luteal phase by increasing the concentrations of progesterone and cAMP. Although RARα expression levels decrease in the secretory phase, a certain amount of RARα is necessary in the mid-luteal secretory phase for further decidualization and embryo implantation. The results of decidualized T-HESCs with RARα overexpression (**Figure 4B**) also support this suggestion, although RARα expression decreased in decidualization compared with undifferentiated status, RARα overexpression enhanced decidualization. In the proliferative phase, RARα may be involved in the regulation of other genes related to proliferation. The role of RARα in decidualization should not be ignored only because its declined trend during secretory period.

Although we have outlined a potential mechanism whereby RARα affects decidualization, there are some limitations to the interpretability of our findings. First, this was an *in vitro* study and was therefore only able to capture some of the essential characteristics of the *in vivo* environment. Second, many other genes are potential downstream candidates of RARα. Further studies are necessary to explore other mechanisms of action of RARα beyond the single potential mechanism proposed here.

In conclusion, our results demonstrated that RARα plays an important role in the mid-luteal phase endometrium. In endometrial stromal cells, RARα directly regulates *CEBPB* transcription during decidualization. A deficiency of RARα decreases C/EBPβ expression levels in RIF patients, leading to decidualization defects and, subsequently, impaired embryo implantation.

## Data Availability Statement

The raw data supporting the conclusions of this article will be made available by the authors, without undue reservation.

## Ethics Statement

The studies involving human participants were reviewed and approved by Institutional Review Board of Center for Reproductive Medicine, Shandong University. The patients/participants provided their written informed consent to participate in this study.

## Author Contributions

CH designed and performed the study, also analyzed data and drafted the manuscript. QZ and TN collected the clinic samples. TZ and CL performed part of real-time PCR and immunochemistry. YL helped improving the study design. JY and Z-JC planned and supervised the study. All authors have approved the final version.

## Funding

This study was supported by National Key Research & Development Program of China (2018YFC1002804, 2016YFC1000202).

## Conflict of Interest

The authors declare that the research was conducted in the absence of any commercial or financial relationships that could be construed as a potential conflict of interest.

## Publisher’s Note

All claims expressed in this article are solely those of the authors and do not necessarily represent those of their affiliated organizations, or those of the publisher, the editors and the reviewers. Any product that may be evaluated in this article, or claim that may be made by its manufacturer, is not guaranteed or endorsed by the publisher.
